# Combined Liposome–Gold Nanoparticles from Honey: The Catalytic Effect of Cassyopea^®^ Gold on the Thermal Isomerization of a Resonance-Activated Azobenzene

**DOI:** 10.3390/molecules29173998

**Published:** 2024-08-23

**Authors:** Guido Angelini, Carla Gasbarri

**Affiliations:** Department of Pharmacy, University “G. d’Annunzio” of Chieti-Pescara, via dei Vestini, 66100 Chieti, Italy; carla.gasbarri@unich.it

**Keywords:** sustainability, honey, gold nanoparticles, liposomes, catalytic activity, 4-methoxyazobenzene, cis–trans isomerization, surface charge

## Abstract

Gold nanoparticles (AuNPs) have been synthesized directly inside liposomes using honey as a reducing agent. The obtained aggregates, named Cassyopea^®^ Gold due to the method used for their preparation, show remarkable properties as reactors and carriers of the investigated AuNPs. A mean size of about 150 nm and negative surface charge of -46 mV were measured for Cassyopea^®^ Gold through dynamic light scattering and zeta potential measurements, respectively. The formation of the investigated gold nanoparticles into Cassyopea^®^ liposomes was spectroscopically confirmed by the presence of their typical absorption band at 516 nm. The catalytic activity of the combined liposome–AuNP nanocomposites was tested via the thermal cis–trans isomerization of resonance-activated 4-methoxyazobenzene (MeO-AB). The kinetic rate constants (*k*_obs_) determined at 25 °C in the AuNP aqueous solution and in the Cassyopea^®^ Gold samples were one thousand times higher than the values obtained when performing MeO-AB cis–trans conversion in the presence of pure Cassyopea^®^. The results reported herein are unprecedented and point to the high versatility of Cassyopea^®^ as a reactor and carrier of metal nanoparticles in chemical, biological, and technological applications.

## 1. Introduction

The development of nanostructures in the range of 1–100 nm through the direct control of atoms and molecules represents the main goal of nanotechnology. Remarkable advances in different fields, such as material science, drug delivery, and agriculture, have been made in recent decades, inspired by Prof. Richard Feynman and his celebrated lecture *There’s Plenty of Room at the Bottom*, which was given in 1959; here, the concept of nanoscale machines was introduced for the first time, as was the possibility of manipulating particles structures during their synthesis [1,2,3,4,5]. Great attention is currently being paid to the design and application of metal nanoparticles, especially in the case of gold (AuNPs) and silver (AgNPs) nanoparticles due to their unique biological, chemical, and physical properties [6,7,8,9]. In addition to their high versatility and stability, AuNPs and AgNPs exhibit antibacterial, antiviral, antineoplastic, and anti-inflammatory activities and play a fundamental role in a large series of devices [10,11,12]. Generally, the biological and chemical properties of gold and silver nanoparticles depend on their size and shape, which are in turn closely linked to the method used for their synthesis. The presence of AuNPs and AgNPs in a solution is confirmed by the appearance of a typical surface plasmonic absorption band in the UV–vis spectrum, whose wavelength and amplitude are indicative of the NPs dimensions and morphology.

Chemical and physical approaches are most commonly used for the preparation of metal nanoparticles. The former require reducing, stabilizing, and coating reagents that have to be removed as contaminants after the reaction. The latter offer the advantage of avoiding chemicals but require more expensive conditions; for example, the pressure and temperature may need to be modulated, and this process may consume a lot of energy. Recently, green approaches have been proposed, starting from natural compounds such as microorganisms, plant extracts, or organic sources [13,14,15,16]. The use of honey represents one of the most sustainable, reproducible, and fast methods for obtaining highly monodisperse and stable metal nanoparticles with controlled biological properties and size. The honey-assisted synthesis of silver and gold nanoparticles occurs through the reduction of Ag^+^ and Au^3+^ ions into metallic Ag^0^ and Au^0^, respectively, without the addition of chemicals as stabilizers and coating agents [17,18,19]. Previously, the honey-assisted synthesis of silver nanoparticles was performed in aqueous solution and inside supramolecular aggregates composed of a natural phospholipid used as a reactor and delivery system; this was named Cassyopea^®^, acronym of ***c**ombined **a**nd **s**ustainable **sy**nthesis **o**f **p**ayload-**e**nriched **a**ggregates*, from the method used for their preparation. The formation of the investigated AgNPs, named NewAgNPs^®^, into Cassyopea^®^ was spectroscopically and kinetically demonstrated. The obtained liposome–AgNP nanocomposites, with a mean size of 138 nm and a surface charge of about −69 mV, remain stable for at least 9 months. Interestingly, it was observed that Cassyopea^®^ liposomes protect metallic silver from the oxidation induced by H_2_O_2_ and preserve its antibacterial activity against *E. coli* and *P. aeruginosa*, selected as Gram-negative bacteria, and against *S. aureus* and *B. cereus*, selected as Gram-positive bacteria [20]. Recently, it was observed that the combination of liposomes and gold nanoparticles can achieve signal amplification for biosensors, especially for theragnostic applications [21,22,23,24]. Gold nanoparticles exhibit unique optical properties, as widely demonstrated by different applications, based on their interactions with light, including medical imaging, diagnostics, biosensing, and photothermal therapy [25,26,27,28]. Their use as catalysts for a large series of reactions has also been confirmed by adding a protective agent, such as polyvinyl alcohol, polyvinylpyrrolidone, or cyclodextrins, to control the nanoparticles’ size and shape [29,30].

The aim of this work was to prepare and characterize Cassyopea^®^ Gold liposomes consisting of supramolecular aggregates used as a reactor and carrier for the synthesis and delivery of AuNPs from honey. The gold nanoparticles were created directly inside liposomes under eco-friendly conditions rather than through loading them in a preformed state. In previous work, the association between liposomes and AuNPs has been described in depth with regard to their preparation, architecture, and properties [31]. In particular, it was observed that gold nanoparticles can be inserted between phospholipids, incorporated in the inner liposomal core, or absorbed onto their membrane surface according to experimental conditions.

To the best of our knowledge, the reduction of Au^3+^ salt by honey during the formation of large unilamellar vesicles has not been previously performed. The presence of the investigated AuNPs in Cassyopea^®^ liposomes and their location were investigated through Cryo-TEM analysis. The surface charge and the mean size of the Cassyopea^®^ Gold were determined through dynamic light scattering and zeta potential measurements. Finally, their catalytic activity was kinetically demonstrated through the thermal cis–trans isomerization of 4-methoxyazobenzene (MeO-AB), as a model reaction, and compared to the data obtained from the investigated AuNPs in aqueous solution.

## 2. Results and Discussion

**Figure 1:**
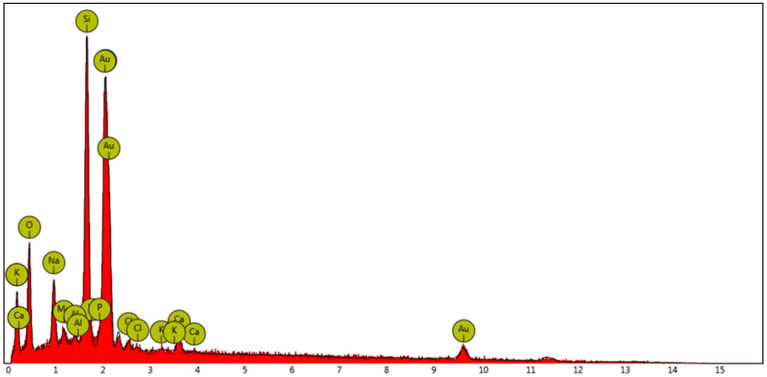
The EDX spectrum of the investigated AuNPs obtained in aqueous solution by using honey as a reducing agent.

Generally, the color and shade of gold nanoparticles in an aqueous solution vary from pink to purple according to their concentration and dimensions [32]. Moreover, a typical surface plasmonic resonance band around 500 nm in the UV–vis spectrum is indicative of the presence of spherical nanoparticles [17]. The efficacy of Acacia honey as a reducing agent for the conversion of gold ions into gold nanoparticles was first verified in the absence of liposomes, as described in the Experimental Section. Gold nanoparticles with a mean size of about 20 nm and a surface charge of −22 mV were obtained in aqueous solution, as determined through dynamic light scattering and a zeta potential analysis (Figures S1 and S2). The formation of the AuNPs was also confirmed through energy dispersive X-ray (EDX) and UV–vis spectroscopy. The strong peak around 2 keV observed in the EDX spectrum is typical for the absorption of metallic gold nanoparticles, alongside the plasmonic absorption band at 520 nm detected in the UV–vis spectrum (Figure S3). The EDX spectrum of the investigated AuNPs is shown in Figure 1.

**Figure 2:**
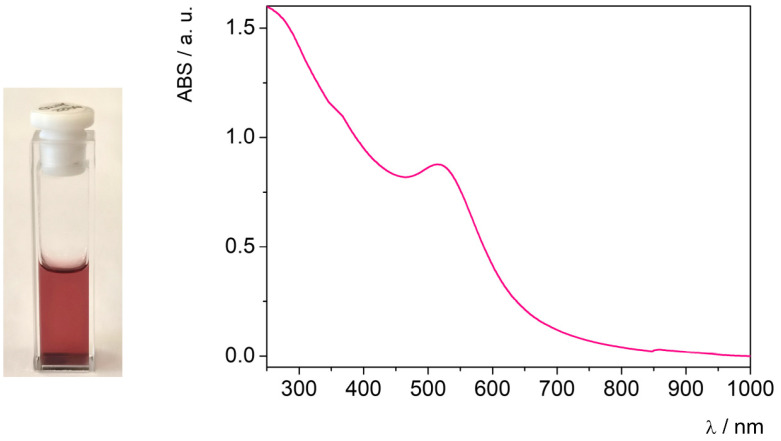
UV–vis spectrum of the Cassyopea^®^ Gold solution at 25 °C.

AuNP synthesis through the reduction of Au^3+^ ions into metallic Au^0^, promoted by honey in aqueous solution, was also successfully performed in Cassyopea^®^ liposomes at room temperature. The resulting Cassyopea^®^ Gold solution was characterized by an intense ruby-red color and by the presence of the plasmonic absorption band at 516 nm in the UV–vis spectrum (Figure 2).

The size, polydispersity, and surface charge of Cassyopea^®^ Gold were determined through dynamic light scattering and a zeta potential analysis. The results are reported in Table 1.

**Figure 3:**
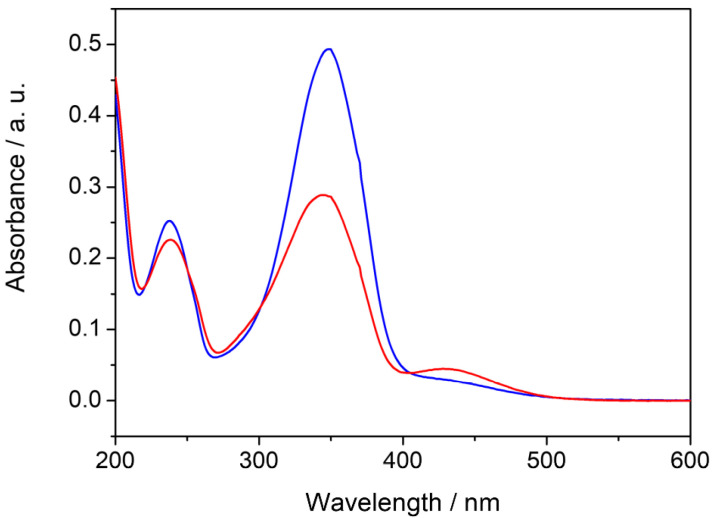
UV–vis spectra of MeO-AB added to the Cassyopea^®^ Gold solution before (blue line) and after (red line) irradiation.

It is well known that the polydispersion index (PdI) provides the particle size distribution within the populations of a given colloidal solution. The PdI falls in the range 0–1 and is closer to 0 in the case of monodisperse particles that are highly homogeneous in size. The surface charge represents a useful parameter for predicting stability over time, since the absence of agglomeration or flocculation phenomena depends on the degree of electrical repulsion on the nanoparticle net surface [33,34]. The highly homogeneous size and the low polydispersion index obtained in the case of Cassyopea^®^ Gold suggest the presence of supramolecular aggregates that are mainly monodispersed in aqueous solution. Furthermore, the zeta potential of −46.5 mV agrees with the negative values observed in the case of phosphatidylcholine liposomes and indicates a low tendency toward self-aggregation [35,36]. Since the catalytic activity and properties of unsupported AuNPs have been previously examined in several model reactions including the cis–trans isomerization of azobenzenes [37,38], the role of Cassyopea^®^ Gold as a catalyst has been tested through the cis–trans isomerization of the 4-methoxy-azobenzene molecule, an azobenzene derivate that is very reactive due to the presence of an electron-donating group in its *para* position. Azobenzenes are versatile light-sensitive compounds that have been extensively studied for decades and widely employed in photo-switchable devices [39,40]. The photoisomerization of the *trans* isomer into its *cis* form occurs under UV irradiation, while the reverse conversion takes place spontaneously in the dark due to the lower stability of the *cis* isomer. This thermal cis–trans isomerization can be spectrophotometrically followed, and first-order kinetic rate constants can be measured in different media [41,42,43]. Interestingly, the mechanism by which the reaction occurs has still not been unequivocally established, due to the competition between rotation and inversion identified by typical V-shaped Hammett plots for azobenzene and its derivatives due to the solvent properties and the electron nature of its substituents. It was observed that, in the case of resonance-activated 4-methoxyazobenzene, thermal cis–trans isomerization occurs only through the rotation mechanism in a series of organic solvents, room-temperature ionic liquids, and micellar solutions and in the presence of metal nanoparticles [44,45,46,47,48]. Interestingly, the reaction of the nitrogen–nitrogen π-bond breakdown and the increase in the electron density in the π* orbital of the molecule due to the mesomeric effect of the methoxy group in the *para* position are responsible for the formation of the highly dipolar transition state, which significantly enhances the rate of the process in comparison to that of azobenzene and other mono-substituted derivatives. In this work, 4-methoxyazobenzene was added to the investigated samples before and after the irradiation of the MeO-AB ethanolic stock solution, as described in the Experimental Section. The UV–vis spectra of the MeO-AB molecule before and after irradiation in the Cassyopea^®^ Gold samples are reported in Figure 3.

**Figure 4:**
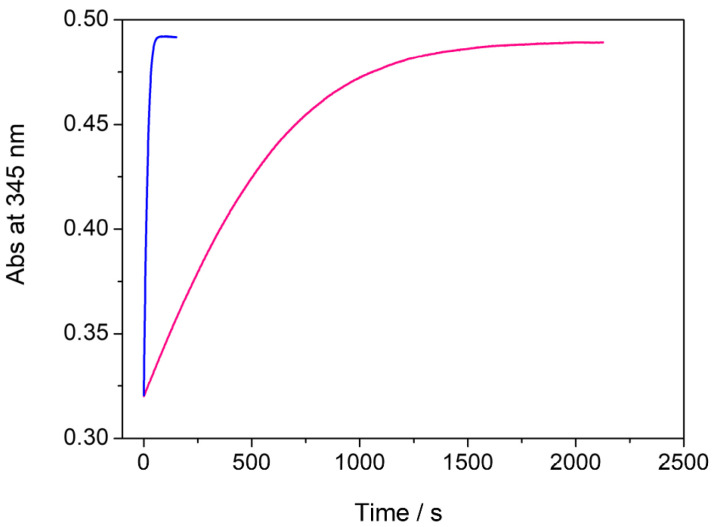
Comparison between the kinetic profile of the cis–trans isomerization of MeO-AB in the presence of AuNPs (blue color) and in the presence of Cassyopea^®^ Gold solution (pink color), as determined at 25 °C.

It was assumed that thermodynamically stable *trans* MeO-AB is the prevailing isomer in the solution before its exposure to UV light [49]. Its conversion into the *cis* isomer was confirmed by typical spectral changes, such as a reduction in the high-intensity π → π* transition absorption band centered around 345 nm and a contemporary but not proportional increase in the broad low-intensity n → π* transition absorption band around 440 nm. The cis–trans isomerization of the MeO-AB molecule in aqueous solution in the presence of the Cassyopea^®^ Gold liposomes was determined at 25° C in the dark by monitoring the increase in the absorption maximum of the *trans* isomer at 345 nm over time, according to its first-order decay. Examples of the kinetic profile of the cis–trans isomerization of 4-methoxyazobenzene in the presence of AuNPs and in the presence of Cassyopea^®^ Gold solution are compared in Figure 4.

The reaction was also performed in the presence of pure Cassyopea^®^ (Figure S6). The first-order rate constants (*k*_obs_) determined for each sample are shown in Table 2.

The thermal cis–trans isomerization of azobenzenes tends to be a very slow process, and in some cases, more days are needed to complete the reaction [50]. Typical *k*_obs_ in the order of 10^−6^ s^−1^ were determined at 25 °C for a series of azoderivatives, including MeO-AB, in a series of organic solvents and room-temperature ionic liquids, and in aqueous solution with silver nanoparticles. In particular, a *k*_obs_ of 3.66 × 10^−6^ s^−1^ was measured in ethanol solution; a *k*_obs_ of 2.77 × 10^−6^ s^−1^ was determined in methanol solution; a *k*_0_ of 2.11 × 10^−6^ s^−1^ was extrapolated in an aqueous solution made from methanol–water mixtures due to the low solubility of MeO-AB; finally, *k*_obs_ values of 3.90, 3.42, and 3.54 × 10^−6^ s^−1^ were obtained in neutral, anionic, and cationic micellar solutions, respectively [47]. A similar trend was observed in a series of imidazolium room-temperature ionic liquids, such as BMIM PF_6_, BMIM BF_4_, BMIM Tf_2_N, EMIM Tf_2_N, BM_2_IM Tf_2_N, and HMIM Tf_2_N. It was observed that the *k*_obs_ value measured at 25 °C changes from a maximum of 22.7 × 10^−6^ s^−1^ in BMIM BF_4_ to 6.83 and 4.19 × 10^−6^ s^−1^ in BMIM PF_6_ and BMIM Tf_2_N, respectively. Interestingly, this kinetic behavior was mainly attributed to the different properties of the BF_4_^−^ anion in comparison to Tf_2_N^−^ and PF_6_^−^, such as its lower dispersion charge, higher Kamlet-Taft β value, and smaller van der Waals radius [43]. Furthermore, the thermal isomerization of MeO-AB becomes faster with increasing temperatures; for example, the *k*_obs_ increased by about 108 times in BMIM PF_6_ and 52.5 times in BMIM Tf_2_N when the temperature was increased from 15 to 50 °C. The activation energies of the reaction were also calculated according to the Arrhenius and Eyring equations [44]. Recently, the thermal cis–trans isomerization of MeO-AB was performed in aqueous solution with silver nanoparticles at 25 °C. *k*_obs_ values in the range 2.17–3.02 × 10^−6^ s^−1^ were determined upon increasing the silver nanoparticle concentration in the solution from 0.32 ppm to 32 ppm, and the closeness of the values obtained in the concentrated and diluted samples demonstrated that the reaction rate constant was not affected by the AgNP concentration. Interestingly, in the AgNP sample in which the MeO-AB was left for 120 h in the dark before irradiation, the rate constants for cis–trans isomerization were determined according to a stretched exponential kinetic analysis, suggesting a change in the chemical environment during the reaction [48].

**Figure 5:**
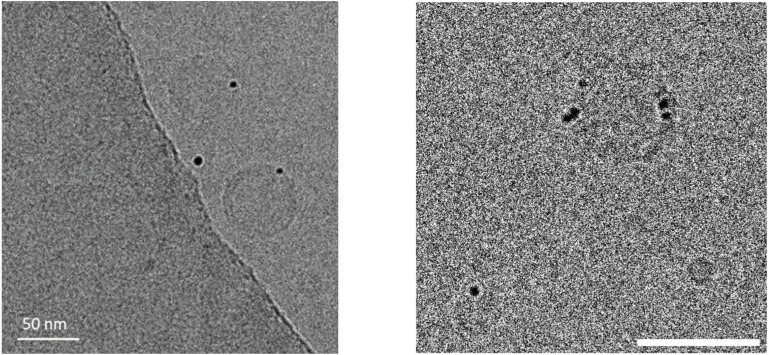
Examples of Cryo-TEM images of Cassyopea^®^ Gold sample. Scale bar corresponds to 50 nm.

In the case of the MeO-AB cis–trans isomerization performed in aqueous solution in the presence of pure Cassyopea^®^ liposomes, a *k*_obs_ of 8.15 × 10^−6^ s^−1^ was determined (Table 2). This value is about four times higher than the *k*_0_ extrapolated in aqueous solution using methanol–water mixtures, as previously reported [47], and this result could be due to the interaction between the azobenzene derivate and the liposomal membrane. It is well known that the *trans* and the *cis* isomers of *para*-substituted azobenzenes strongly differ in their ability to interact with phospholipid membranes. In particular, the former is less interactive than the latter, which tends to insert into the bilayer better due to its non-planar geometry. Moreover, it was demonstrated that *cis* MeO-AB promotes more intense interactions with negatively charged membranes due to its larger dipole moment in comparison to its *trans* isomer. It was calculated that the molecular change in the geometry of MeO-AB during its *trans* to *cis* conversion state decreases the distance between the 4-4′ positions of the azobenzene moiety and consequently enhances the dipole moment from 1.86 to 4.54 Debye [47,51]. The reaction rate of this cis–trans isomerization is strongly enhanced in the presence of the investigated AuNPs, as demonstrated by the corresponding *k*_obs_ value, which becomes one thousand times higher in comparison to the *k*_obs_ previously observed without AuNPs. *k*_obs_ values of 69.3 × 10^−3^ and 1.80 × 10^−3^ s^−1^ were determined in the AuNP aqueous solution and in Cassyopea^®^ Gold, respectively. The role of gold nanoparticles as a catalyst for the cis–trans conversion of the MeO-AB molecule has been previously described [38]. Faster isomerization was observed for the 4-methoxyazobenzene derivate in comparison to other *para*-substituted azobenzenes, and this behavior was attributed to the remarkable proximity between the azo molecule and the metal surface, promoted by the strong dipole moment of the *cis* MeO-AB, which improves the electron transfer involved in the reaction. In the case of Cassyopea^®^ Gold liposomes, the catalytic activity of the investigated AuNPs in aqueous solution was maintained but their cis–trans isomerization occurred about 38 times more slowly. This effect suggests that the electron transfer between the 4-methoxyazobenzene molecule and the metal surface on which the catalytic activity of the AuNPs depends is slowed down due to the incorporation of the investigated gold nanoparticles into the phospholipid bilayer of Cassyopea^®^. Recent studies based on the electrostatic and van der Waals interactions between citrate-stabilized gold nanoparticles and large unilamellar vesicles made with zwitterionic phospholipids have demonstrated the size-dependent behavior of AuNPs added to a liposome solution in a preformed state. It was demonstrated that nanoparticles with dimensions in the range of 5–10 nm tend to aggregate on the membrane surface or be engulfed as wrapped linear aggregates within a tubular membrane, that AuNPs in the range of 25–35 nm are generally adsorbed on the surface with partial wrapping, and finally that larger nanoparticles with a mean size over 50 nm are adsorbed onto the liposomal surface and able to promote membrane bending up to remarkable penetration depths [52]. In order to deeply investigate the distribution of the investigated AuNPs during the formation of the Cassyopea^®^ Gold liposomes, a Cryo-TEM analysis of the sample was carried out. Examples of micrographs from the investigated sample are reported in Figure 5.

Spherical and unilamellar vesicle-loading gold nanoparticles can be observed. Interestingly, the mean size of about 20 nm measured for the AuNPs through dynamic light scattering seems to differ from the mean size of about 5 nm observed for the same nanoparticles in the Cryo-TEM images of Cassyopea^®^ Gold. The dimensions indicated by the scattering analysis actually take into account the self-aggregation of the nanoparticles in aqueous solution due to their negative surface charge of about −22 mV, as determined by the zeta potential analysis. Furthermore, it can be observed that the investigated AuNPs obtained from honey during the formation of Cassyopea^®^ Gold tend to be located in the lipid membrane rather than inside the inner liposomal core, as previously described for gold nanoparticles from citrate obtained in liposomes during their hydration [53].

## 3. Conclusions

In this work, Cassyopea^®^ Gold liposomes derived from the combination of large unilamellar vesicles and gold nanoparticles, obtained using honey as a precursor, were investigated using a sustainable method. The AuNPs were synthesized directly into liposomes in aqueous solution and at room temperature. The Cassyopea^®^ Gold liposomes were characterized in terms of their dimensions, polydispersity, surface charge, and catalytic activity. A mean size of about 150 nm and a negative surface charge of about −46 mV were determined through dynamic light scattering and zeta potential measurements, respectively. All the data indicate high stability, reproducibility, and sustainability. Interestingly, the investigated negatively charged gold nanoparticles with a mean diameter of about 5 nm show a tendency to insert themselves into the bilayer of the Cassyopea^®^ liposomes, as demonstrated by the Cryo-TEM analysis and confirmed by following the reaction of the thermal cis–trans isomerization of 4-methoxyazobenzene, added as a *cis* isomer to the samples. To the best of our knowledge, the method and data described herein to generate AuNPs within liposomes from honey are unprecedented. These results, as well as those previously obtained for the synthesis of silver nanoparticles from honey, confirm the wide versatility and remarkable properties of Cassyopea^®^ as a reactor and carrier and show its promising role in the field of nanotechnology.

## 4. Experimental Section

### 4.1. Materials

Gold (III) chloride trihydrate (purity ˃ 99.9%), 1-palmitoyl-2-oleoyl-phosphatidylcholine (POPC) (purity ˃ 99%), and ethanol (99% spectroscopic grade) were purchased from Sigma-Aldrich (Milan, Italy). Italian Acacia honey was purchased directly from a beekeeper and used without further purification. 4-methoxyazobenzene (MeO-AB) was synthesized as previously described [47]. All the samples were diluted with Milli-Q (Merk, Milan, Italy) water.

### 4.2. Instruments

The spectroscopic analysis was performed by transferring 2 mL of the Cassyopea^®^ Gold aqueous solution into 1 cm light-path quartz cuvettes. The UV–vis spectra were determined at 25 ± 0.1 °C by using a Jasco V-570 spectrophotometer (Jasco corporation, Tokyo, Japan).

The dynamic light scattering analysis and zeta potential measurements were carried out at 298 K by transferring 2 mL of the Cassyopea^®^ Gold aqueous solution into disposable 1 cm light-path plastic cells. The scattering data were extrapolated by using the Stokes–Einstein relationship for the calculation of the hydrodynamic radius with a Brookhaven (90PLUS BI-MAS, Nasha, NH, USA) digital correlator at a scattering angle of 90°. The instrument was equipped with a 35 mW He–Ne laser at a wavelength of 660 nm. For the zeta potential data, an angle of 15° instead of 90° was employed.

### 4.3. Preparation of the Cassyopea^®^ Gold Samples

The AuNPs were synthesized by gradually mixing an aliquot of HAuCl_4_ 0.002 M with an aliquot of 1% Acacia honey in aqueous solution at a basic pH under stirring at room temperature until the color of the solution turned to an intense ruby red. The Cassyopea^®^ liposomes used in this work consisted of large unilamellar POPC vesicles prepared through phospholipidic film hydration, followed by extrusion according to a previously described method [20]. In brief, an aliquot of a 13 mM organic POPC solution was placed in a flask and the organic solvent was removed by evaporation under vacuum to obtain a dry film; this was used as a reaction medium for the gold nanoparticles’ synthesis, as described above. The investigated AuNPs were generated directly inside the liposomes, not loaded in a preformed state. The gold and POPC concentrations in a Cassyopea^®^ sample diluted to a neutral pH corresponded to 4 × 10^−5^ M and 1.3 × 10^−4^ M, respectively.

### 4.4. Kinetics Measurements

A stock ethanol solution of 4-methoxyazobenzene was prepared at a concentration of 2.6 × 10^−3^ M and kept in the dark at room temperature for at least 4 days before use. Then, an appropriate amount was transferred into a 1 cm light pass quartz cuvette and irradiated for 90 min using a Hg–Xe arc lamp (200 W) equipped with a band-pass interference filter centered at a 365.0 +2/−0 nm wavelength and 10.0 +2/−2 nm bandwidth. The decrease in the high-intensity absorption band at 345 nm (due to the π → π* transition) and the increase in the low-intensity band at about 440 nm (due to the n → π* transition) were used as evidence of the *trans*–*cis* photoisomerization. An aliquot of the irradiated solution was added to a 1 cm light path quartz cuvette containing 2 mL of the following samples: Cassyopea^®^ Gold aqueous solution (i); pure Cassyopea^®^ without AuNPs (ii); and AuNPs without Cassyopea^®^ (iii). The thermal cis–trans conversion of MeO-AB followed a first-order decay, and the kinetic rate constants (*k*_obs_) were spectrophotometrically determined at 25 ± 0.1°C by monitoring the absorption change at the maximum wavelength of the *trans* isomer in the dark over time [43]. The final concentration of 4-methoxyazobenzene in each sample was 2.6 × 10^−5^ M.

### 4.5. Preparation of the Sample for the Cryo-TEM Analysis

Sample vitrification was carried out with a Mark IV Vitrobot (Thermo Fisher Scientific, Waltham, MA, USA). As such, 3 μL was applied to a Quantifoil R 1.2/1.3 Cu 300-mesh grid previously glow-discharged at 30 mA for 30’’ in a GloQube. Immediately after sample application, the grids were blotted in a chamber at 4 °C and at 100% humidity and then plunge-frozen in liquid ethane. The vitrified grids were transferred to a Talos Arctica (Thermo Fisher Scientific) operated at 200 kV and equipped with a Ceta 16 M detector (Thermo Fisher Scientific). Images were acquired at a nominal magnification of 73,000×, corresponding to a pixel size of 1.43 Å/pixel, with a defocus of −3.0 μm.

## Figures and Tables

**Table 1:** Size, polydispersity, and zeta potential data of the Cassyopea^®^ Gold sample.

Mean Size (nm)	Polydispersion Index (PDI)	Zeta Potential (mV)
151.4 ± 1.1	0.282 ± 0.005	−46.5 ± 1.9

**Table 2:** First-order kinetic rate constants (*k*_obs_) of the investigated samples in aqueous solution, determined at 25° C.

Sample	*k*_obs/_s^−1^
Cassyopea^®^ Gold	1.80 (±0.1) × 10^−3^
AuNPs	69.3 (±0.1) × 10^−3^
Pure Cassyopea^®^	8.15 (±0.2) × 10^−6^

## Data Availability

Samples of the investigated AuNPs and Cassyopea^®^ Gold are available from the authors on request.

## Supplementary Materials

The following supporting information can be downloaded at https://www.mdpi.com/article/10.3390/molecules29173998/s1: The dynamic light scattering (DLS) and zeta potential analysis of the investigated samples, the UV–vis spectrum of the AuNPs in aqueous solution, and the thermal 4-methoxyazobenzene’s cis–trans isomerization in pure Cassyopea^®^ solution).
