# Supplementary material for: Combined Liposome–Gold Nanoparticles from Honey: The Catalytic Effect of Cassyopea® Gold on the Thermal Isomerization of a Resonance-Activated Azobenzene
Source: Molecules. 2024 Aug 23;29(17):3998. doi: 10.3390/molecules29173998 (PMC11396676; doi:10.3390/molecules29173998)

Supplementary Material

Combined liposomes-gold nanoparticles from honey in aqueous solution. The catalytic effect of Cassyopea® Gold on the thermal isomerization of a resonance-activated azobenzene.

Guido Angelini * and Carla Gasbarri

Department of Pharmacy, University "G. d'Annunzio" of Chieti-Pescara,

via dei Vestini, 66100 Chieti, Italy

* Corresponding author:

guido.angelini@unich.it

phone: +39-0871-3554785

Index

Fig. S1. DLS analysis of AuNPs from honey in aqueous solution.

Fig. S2. Zeta potential analysis of AuNPs from honey in aqueous solution.

Fig. S3. UV-vis spectrum of AuNPs from honey in aqueous solution.

Fig. S4. DLS analysis of Cassyopea® Gold in aqueous solution.

Fig. S5. Zeta potential analysis of Cassyopea® Gold in aqueous solution.

Fig. S6. MeO-Azobenzene *cis-trans* isomerization in pure Cassyopea® aqueous solution.

Fig. S1. DLS analysis of AuNPs from honey in aqueous solution.

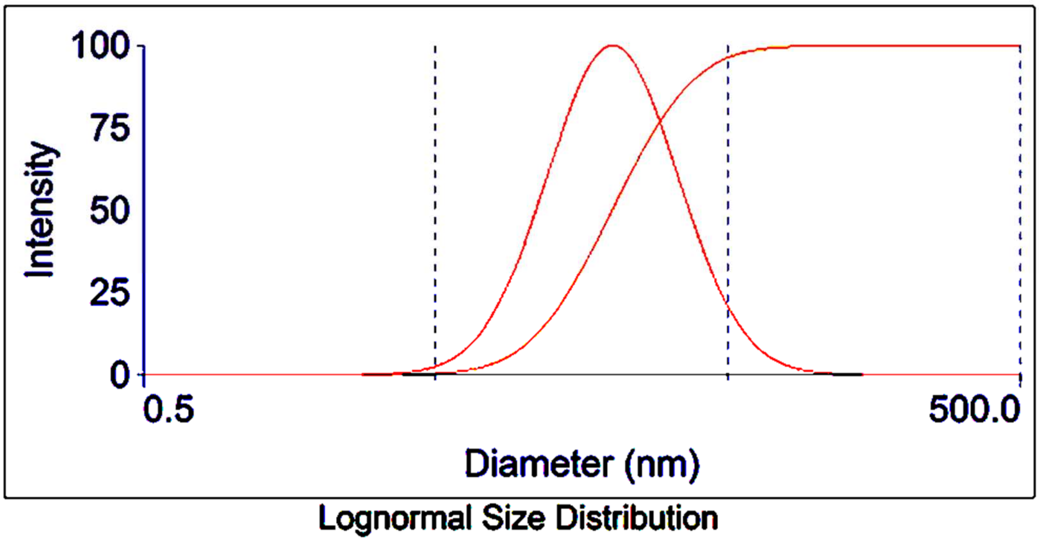

Fig. S2. Zeta potential analysis of AuNPs from honey in aqueous solution.

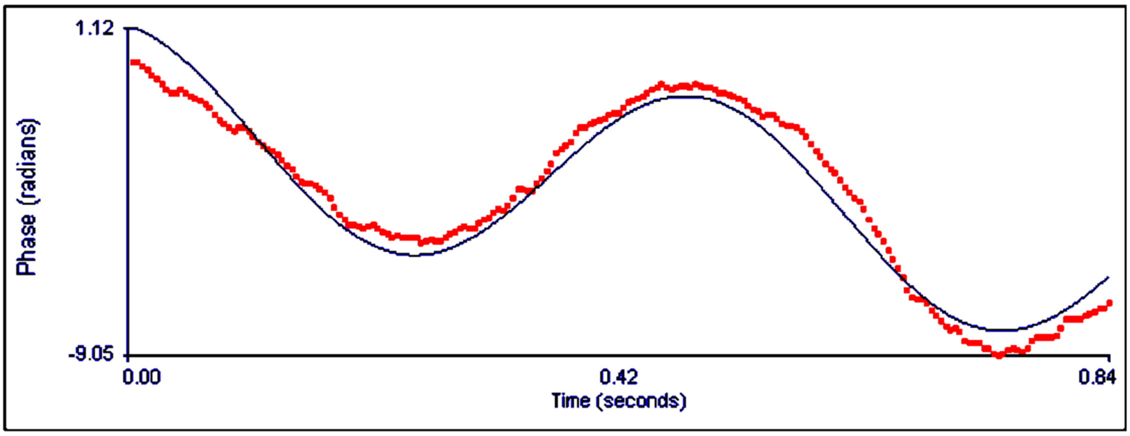

Run	Mobility	Zeta Potential (mV)	Rel. Residual
1	-1.95	-24.94	0.0362
2	-1.12	-14.38	0.0476
3	-1.43	-18.36	0.0902
4	-1.94	-24.81	0.0508
5	-1.79	-22.96	0.0496
6	-2.06	-26.35	0.1075
7	-1.86	-23.76	0.0599
Mean	-1.74	-22.22	0.0631
Std. Error	0.13	1.62	0.0098
Combined	-1.69	-21.60	0.0408

Fig. S3. UV-vis spectrum of AuNPs from honey in aqueous solution.

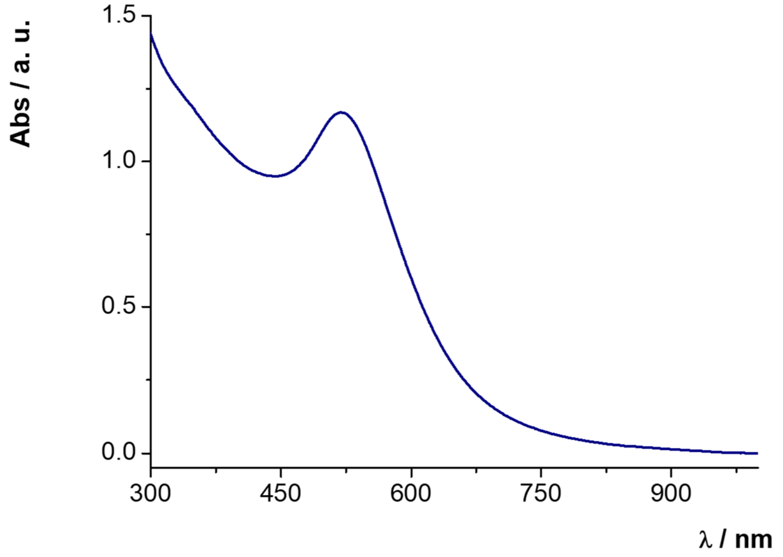

Fig. S4. DLS analysis of Cassyopea® Gold in aqueous solution.

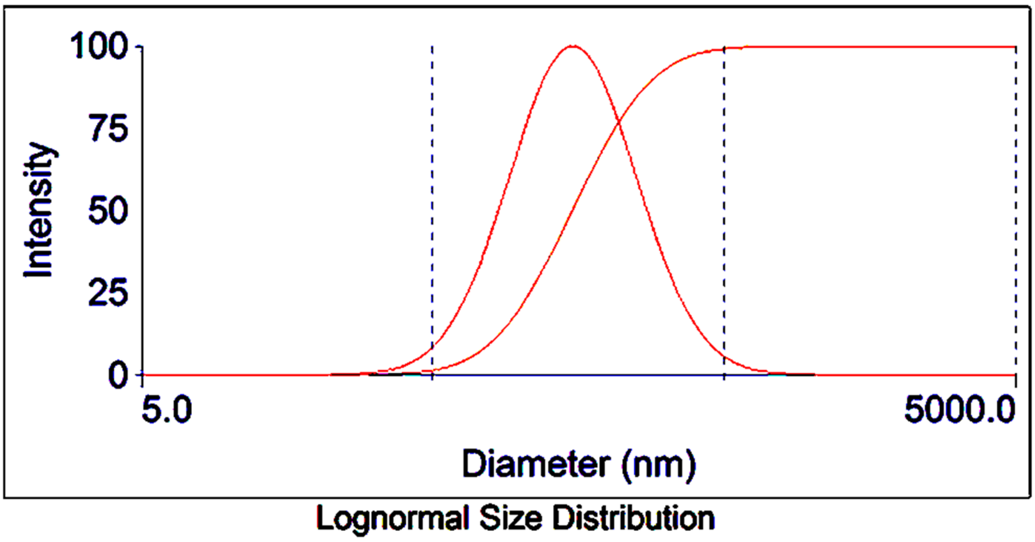

Fig. S5. Zeta potential analysis of Cassyopea® Gold in aqueous solution.

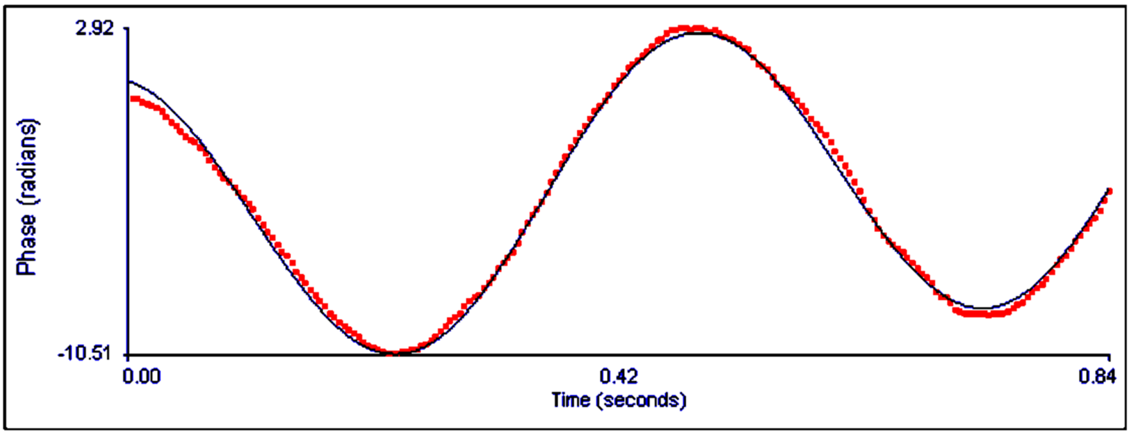

Run	Mobility	Zeta Potential (mV)	Rel. Residual
1	-3.23	-41.35	0.0389
2	-3.57	-45.70	0.0382
3	-4.10	-52.43	0.0389
4	-3.12	-39.89	0.0299
5	-4.14	-52.99	0.0591
6	-3.75	-48.00	0.0408
7	-3.54	-45.34	0.0324
Mean	-3.64	-46.53	0.0397
Std. Error	0.15	1.90	0.0036
Combined	-3.62	-46.35	0.0186

Fig. S6. MeO-Azobenzene *cis-trans* isomerization in pure Cassyopea® aqueous solution.

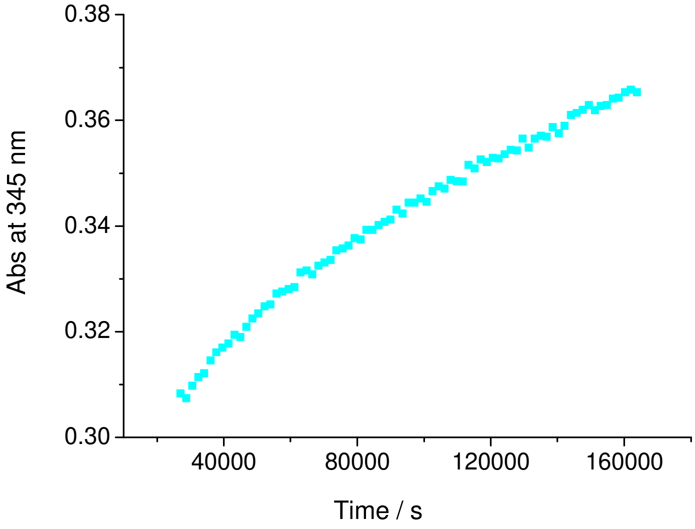

Supplement: Supplementary file 1 [file molecules-29-03998-s001.zip › ANGELINI_molecules-3143733-supplementary.pdf]
